# Renal Cell Carcinoma in Crossed Fused Renal Ectopia: A Case Report of a Rare Congenital Renal Fusion

**DOI:** 10.7759/cureus.108279

**Published:** 2026-05-05

**Authors:** Muniba Manzoor, Muhammad Naveed, Ambreen Sadiq, Muzammel Manzoor

**Affiliations:** 1 Radiology, Combined Military Hospital, Rawalpindi, PAK; 2 Cardiac Surgery, Children's Health Ireland at Crumlin, Dublin, IRL; 3 Radiology, Safari Hospital, Rawalpindi, PAK

**Keywords:** crossed fused ectopia, nephron-sparing, partial nephrectomy, renal cell carcinoma, renal transplant

## Abstract

Crossed fused renal ectopia (CFRE) is a rare congenital renal fusion anomaly in which both kidneys are located on the same side of the body and are usually fused. Simultaneous occurrence of primary renal cell carcinoma (RCC) in CFRE represents a rare entity. Although CFRE may remain asymptomatic, it can be associated with complications such as urinary tract infections, obstruction, calculi, rarely malignancy, and tumors within the fused kidneys, which are exceptionally rare. Although the literature reports only a very small number of cases of CFRE coexisting with RCC, we present a case of a 46-year-old man with CFRE and simultaneous RCC detected on contrast-enhanced computed tomography. Radiological imaging demonstrated that the right kidney was ectopically located in the left iliac region, inferior to the left kidney, with a fused upper pole. Further, a CFRE with a large necrotic renal tumor involving both kidneys was also observed, which suggests RCC within a fused ectopic kidney. Upon these radiological features, total nephrectomy of fused kidneys, hemodialysis afterward, and then renal transplant was decided.

## Introduction

Crossed fused renal ectopia (CFRE) represents the second most common renal fusion anomaly after a horseshoe kidney [1]. It is a rare congenital renal anomaly in which one kidney crosses the midline and fuses with the contralateral kidney, resulting in both kidneys lying on the same side of the abdomen [2,3]. This condition often remains clinically silent but carries important clinical implications due to aberrant ureteric and vascular anatomy [3], with an approximate incidence ranging from one in 2,000 to one in 7,500 cases observed at autopsy, with a predominance of a male-to-female ratio of 1.4 to 2: 1, and has a two to three times higher chance of having a left-to-right ectopy [4,5]. Several anatomical variants have been described, with CFRE being the most common variant, in which the ectopic kidney lies inferiorly and is fused to the orthotopic kidney [6]. The condition is frequently asymptomatic and often discovered incidentally during imaging studies. However, abnormal renal rotation, aberrant vascular supply, and ureteral course may predispose patients to complications including hydronephrosis, infection, and nephrolithiasis [7,8]. In addition, an ectopic kidney is also often associated with other abnormalities, like vascular malformation, agenesis, inconsistencies, urinary tract infection, a palpable abdominal mass, genital anomalies, and a high incidence of stone formation [9]. Notably, malignancy occurring in CFRE is rare, with renal cell carcinoma (RCC) being the most frequently reported tumor [10]. The RCC in CFRE poses unique diagnostic and therapeutic challenges due to its atypical renal location, complex vascular anatomy, and fused parenchyma [11].

Due to the rarity of RCC in CFRE, the existing literature is limited to isolated case reports and small case series, and there are no well-established, standardized management guidelines for this condition. Each case report contributes valuable information regarding clinical presentation, imaging findings, surgical approaches, and clinical outcomes. Such reports are essential for improving clinical awareness and guiding decision-making in similar complex case scenarios. In this context, we report a rare case of bilateral RCC arising within a CFRE, emphasizing the role of radiologic diagnosis and preoperative planning.

## Case presentation

A 46-year-old man presented with weight loss and generalized weakness, with no significant past medical history. There were no constitutional symptoms, no family history of similar congenital anomalies, or malignancy. The physical examination was unremarkable. The laboratory findings showed that most hematological parameters were within normal limits, including red blood cell count, hemoglobin, hematocrit, and red cell indices, indicating no evidence of anemia or significant red cell abnormalities. Platelet count was also within the normal range. However, there are notable signs of an inflammatory or systemic response, as reflected by an elevated total leukocyte count with neutrophilia and monocytosis, which may suggest an underlying inflammatory or malignant process. Serum creatinine is mildly elevated, indicating some degree of renal impairment, which is clinically relevant given the underlying renal pathology, while blood urea nitrogen remains within normal limits. Lymphocyte and eosinophil counts are within or close to normal ranges (Table 1).

**Table 1:** Laboratory parameters TLC: total leucocyte count; Hct: hematocrit; MCV: mean corpuscular volume; MCH: mean corpuscular hemoglobin; MCHC: mean corpuscular hemoglobin concentration

Lab test	Value	Reference range	Interpretation
Total red blood cells	4.75 × 10^6^/µL	4.6-6.2 × 10^6^/µL	Normal
WBC count (TLC)	12.91 × 10^3^/µL	4.5-11 × 10^3^/µL	Elevated
Serum creatinine	1.4 mg/dL	0.6-1.3 mg/dL	Elevated
Blood urea nitrogen	17 mg/dL	8-24 mg/dL	Normal
Hemoglobin	15.7 g/dL	13-17.5 g/dL	Normal
Hct	42.5%	41%-53%	Normal
MCV	89.4 fL	80-100 fL	Normal
MCH	33.1 pg	31-37 pg	Normal
MCHC	37 g/dL	31-37 g/dL	Normal
Platelet count	188 × 10^3^/µL	140-440 × 10^3^/µL	Normal
Neutrophils	70%	54%-62%	Elevated
Lymphocytes	21%	25%-33%	Normal
Monocytes	21%	03%-07%	Elevated
Eosinophils	03%	01%-06%	Normal

**Figure 1:**
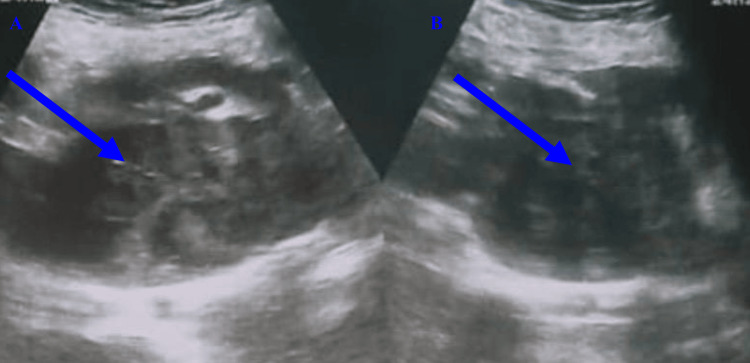
Ultrasound image of the patient (A) Mass showing internal echogenic foci suggesting calcification. (B) Cross-fused kidney in the left iliac fossa showing a heterogeneous, predominantly hypoechoic mass

Ultrasound revealed the absence of the right kidney in the right renal fossa (Figure 1).

**Figure 2:**
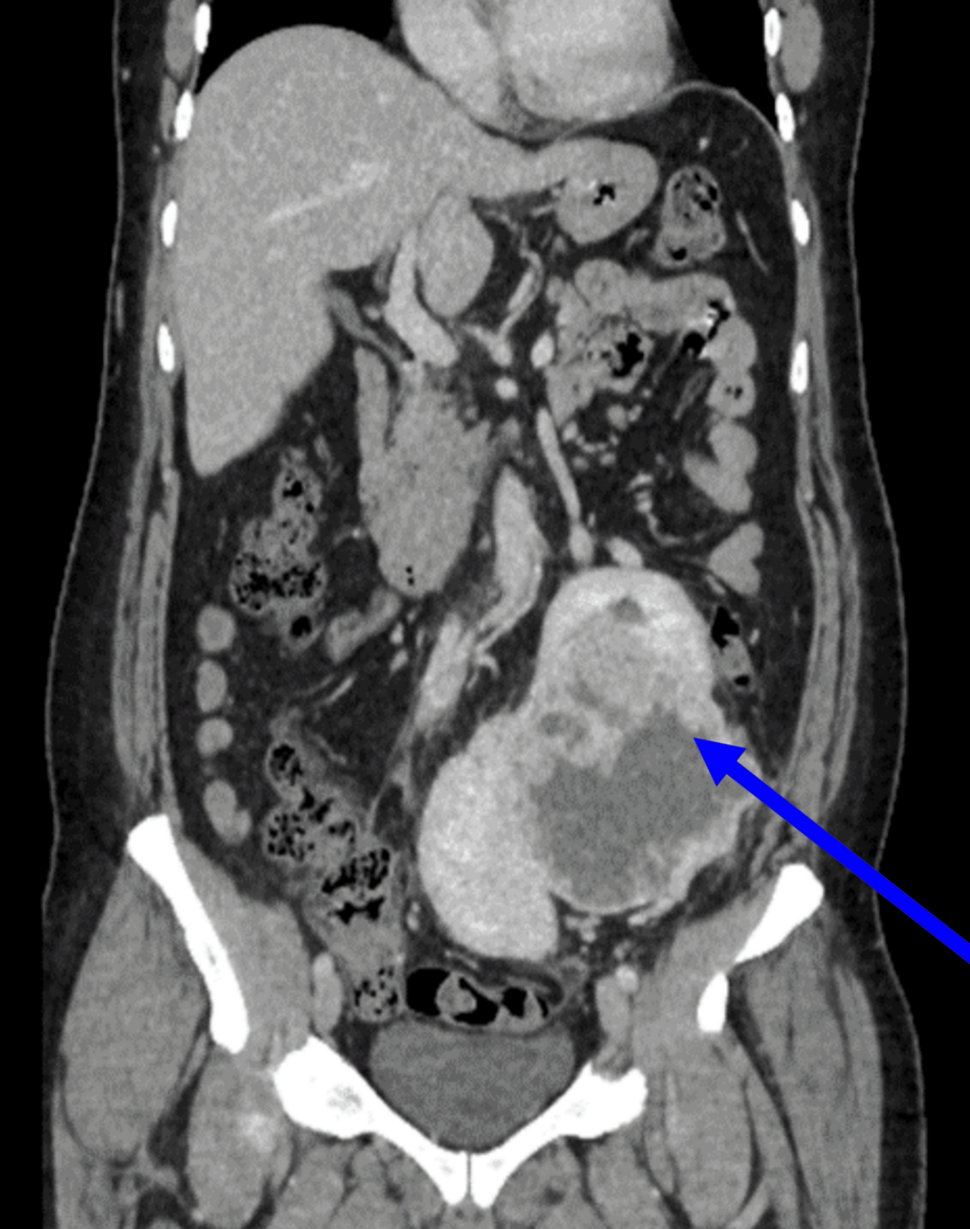
CT abdomen pelvis with a contrast coronal view, showing an empty right renal fossa and an ectopic right kidney fused to the lower pole of the left kidney CT: computed tomography

Two fused renal masses were observed in the left abdomen, with a heterogeneous echogenic lesion in the fused kidneys. For further confirmation, contrast-enhanced computed tomography (CECT) of the abdomen was performed, using an examination technique with 8-mm contiguous slices from the apex to the upper abdomen. Outcomes demonstrated that the right kidney was ectopically located in the left iliac region, inferior to the left kidney, with a fused upper pole (Figure 2).

**Figure 3:**
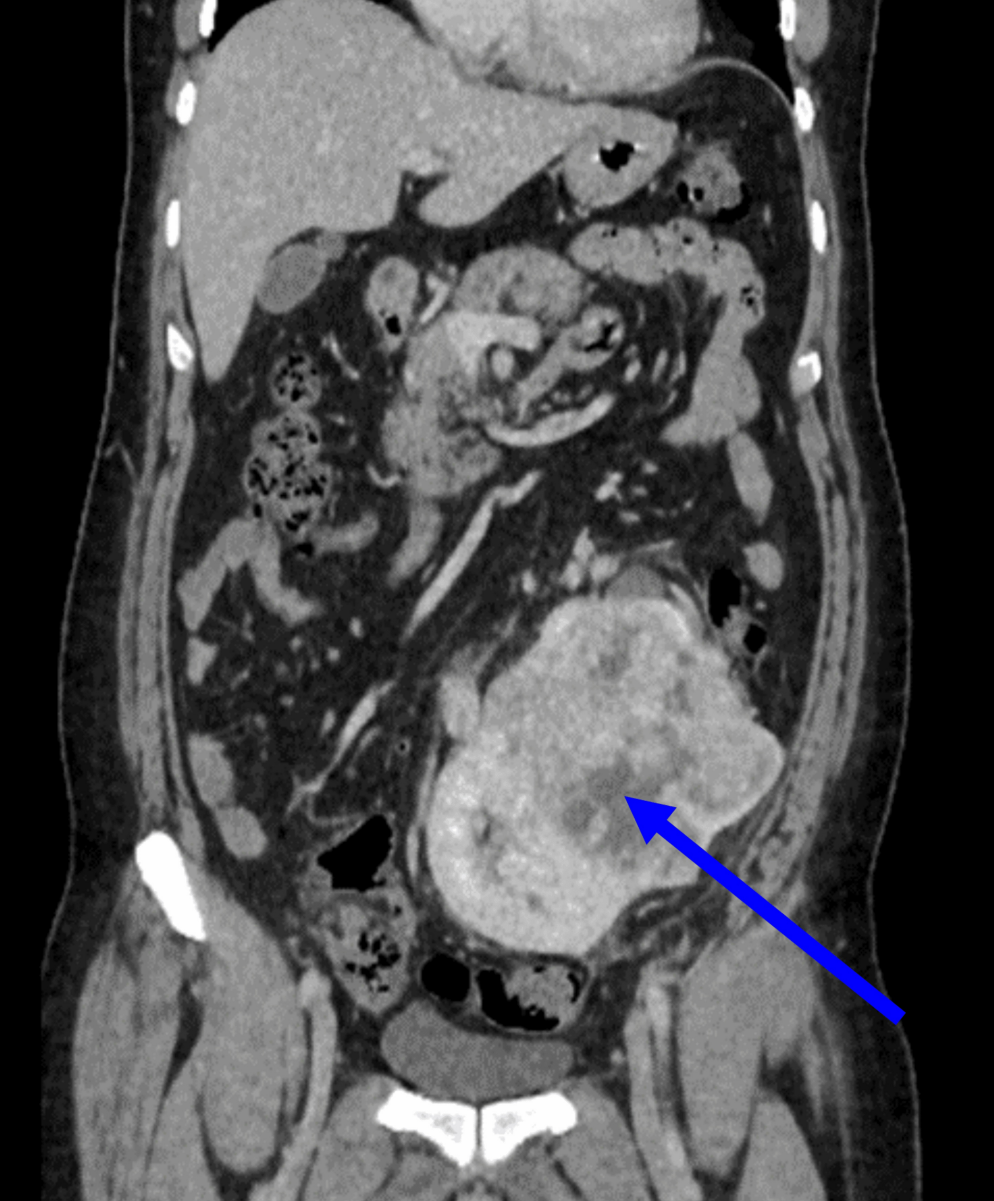
The coronal view showing a heterogeneously enhancing mass involving both renal units

A large mass measuring 12.3 x 11.8 x 6.6 cm (craniocaudal × maximum transverse diameter × anteroposterior) with a central necrotic nonenhancing area was seen involving both kidneys. The pelvicalyceal system of both kidneys was also faced superomedially and stretched by the tumor (Figure 3).

**Figure 4:**
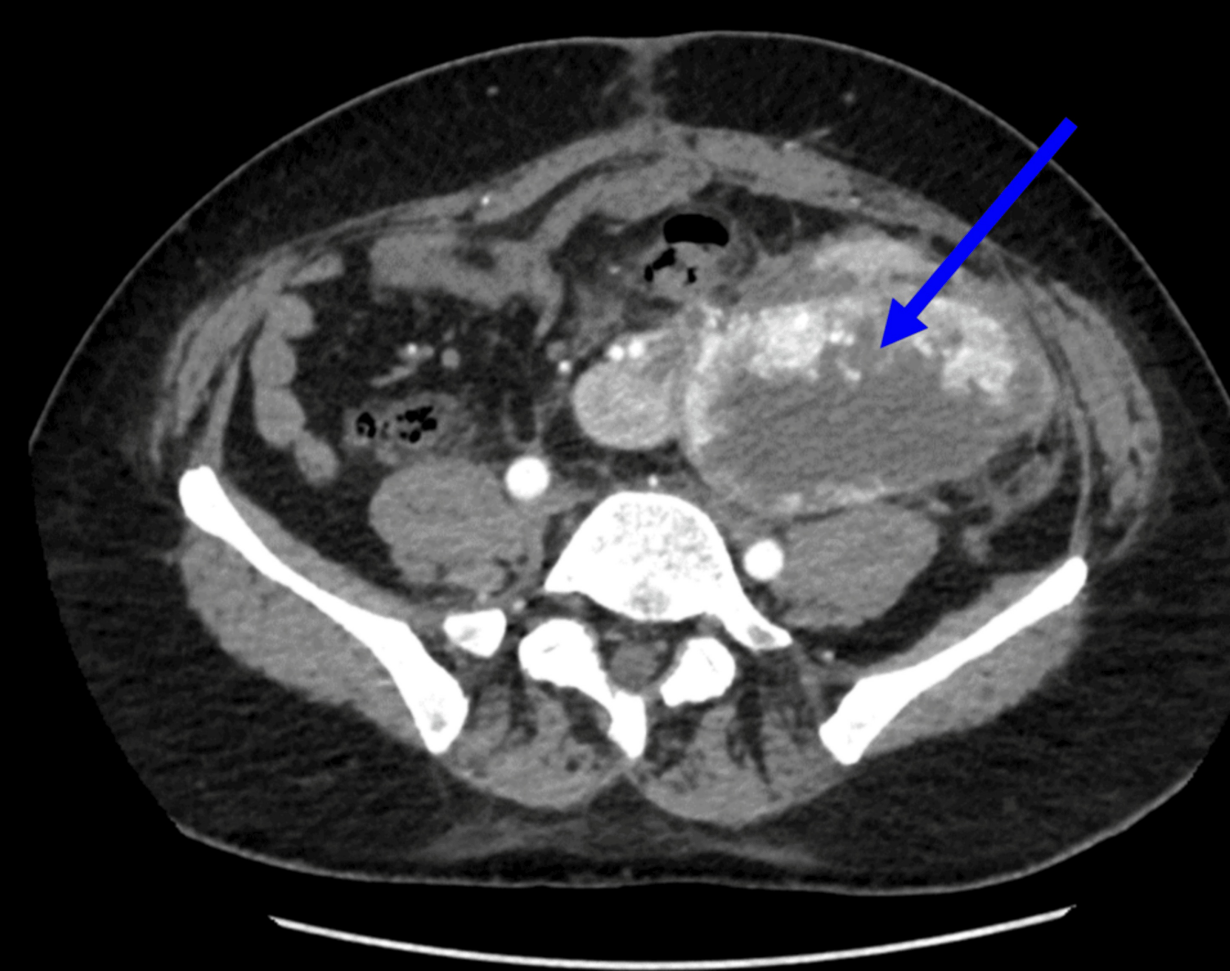
The axial view showing a necrotic component of a renal mass involving both renal units in cross-fussed kidneys

The right ureter crossed the midline and inserted at its normal position in the urinary bladder. The left pelvicalyceal system was dilated, and the left ureter was not outlined. The left kidney is supplied by two renal arteries arising side by side from the aorta; the anterior one supplies the anterior part, and the posterior branch supplies the posterior cortex of the left kidney. The right renal artery was ectopically raised from the distal aorta just above the bifurcation and entered the right kidney. The right renal vein was found to be of normal caliber and entered the right kidney through the hilum. The left renal vein ectopically passed posterior to the aorta and extended to the left renal hilum laterally. Furthermore, necrotic components of the renal mass were also observed (Figure 4).

**Figure 5:**
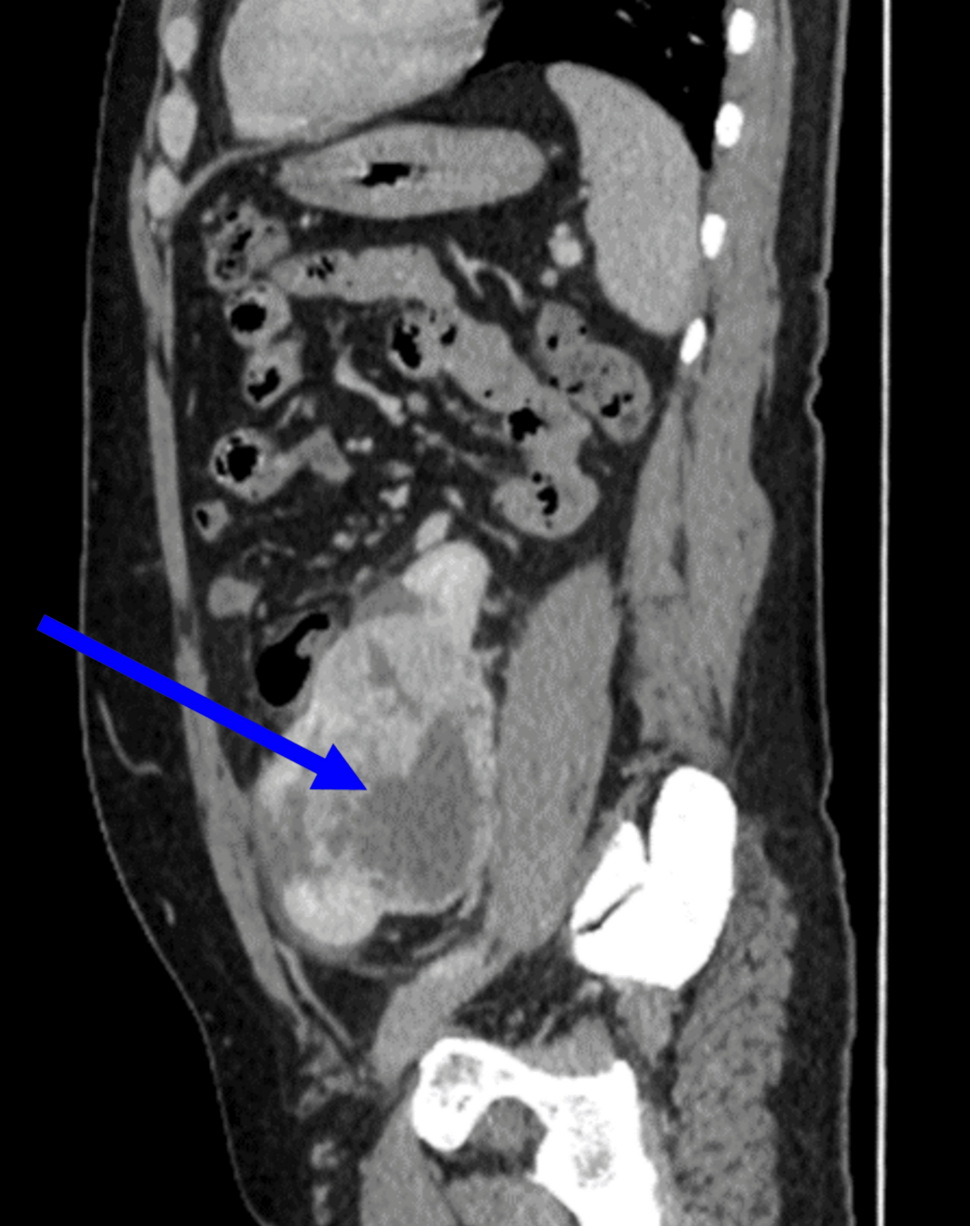
The sagittal image showing an enhancing renal mass involving the lower pole of the left kidney and the upper pole of the cross-fused right kidney

A sagittal image demonstrated an enhanced renal mass involving the lower pole of the left kidney and the upper pole of the cross-fused right kidney (Figure 5). Meanwhile, no renal vein thrombosis or distant metastasis was found. On the basis of these findings, CFRE was found with a large necrotic renal tumor involving both kidneys, LT>RT. Therefore, it is suggestive of RCC within a fused ectopic kidney.

A decision was made to proceed with nephron-sparing surgery with partial nephrectomy of both lesions after detailed preoperative vascular mapping. In this case, more than 50% of both renal units were involved, so in the multidisciplinary team (MDT), it was decided to perform total nephrectomy of fused kidneys, hemodialysis afterward, and then renal transplant. This significantly limited the possibility of preserving adequate functional renal tissue. Additionally, the complex and shared vascular anatomy increased the risk of bleeding, ischemic damage, and incomplete tumor resection with partial nephrectomy. Given these anatomical and oncological challenges, the MDT decided that total nephrectomy would provide better oncological control, with planned postoperative hemodialysis and eventual renal transplantation as the safest management approach. Postoperatively, no complications were observed, and the patient was discharged.

## Discussion

CFRE results from abnormal migration of the metanephric blastema during embryologic development and is a rare, complex congenital renal anomaly. The RCC occurrence within such an anatomical variation is exceedingly uncommon. This coexistence poses unique diagnostic and therapeutic challenges due to its aberrant location and highly variable vascular anatomy, which usually occur during the fourth to eighth gestational weeks [12]. In our case study, these anatomical complexities necessitated careful radiological evaluation and individualized surgical planning to achieve optimal oncological and functional outcomes. Meanwhile, several morphologic types have been described, including the L-shaped kidney, S-shaped or sigmoid kidney, and inferior and superior ectopia [12]. Anomalies, which are frequently related to CFRE, include skeletal abnormalities, imperforate anus, and cardiovascular septal defects [13]. In addition, several theories have been proposed to explain the mechanisms underlying the development of renal fusion anomalies, including the genetic theory, the mechanical theory, the theory of abnormal caudal rotation, and the ureteral theory [14].

For the diagnosis and surgical planning of CFRE, radiologic imaging plays a crucial role, and for its accurate and precise diagnosis, key radiologic features need to be focused on, including the absence of a kidney in one renal fossa, both kidneys located on the same side, fusion of renal parenchyma, malrotation of renal pelvis, aberrant vascular supply, and hydronephrosis [1]. Among imaging modalities, ultrasound is considered a first-line modality and remains a cornerstone for CFRE, particularly in low-resource settings, demonstrating the presence of ectopic fused renal tissue and helping identify a reniform mass across the midline [3]. Numerous studies support its sensitivity in detecting cysts and renal morphology [15-17]. Nevertheless, computed tomography (CT) urography/CECT offers superior delineation of the collecting systems, parenchymal fusion, and vascular anatomy, thereby establishing the anomaly’s exact laterality and type [18,19]. The CFRE diagnosis can also be made with magnetic resonance imaging, which is useful when CT contrast is contraindicated. Additionally, retrograde pyelography can help determine the size and location of stones [20].

Meanwhile, renal tumors arising in CFRE are rare, but most reported cases involve RCC. Due to this rare co-occurrence, only a few cases have described the association, and conclusions regarding the increased risk of malignancy in CFRE remain unclear [21]. This co-occurrence may be attributed to different reasons, including the rarity of CFRE itself, which inherently restricts the population at risk and limits the ability to generate large, robust datasets. Another reason is the asymptomatic nature of CFRE and often undiagnosed unless incidentally detected, leading to potential underreporting of both the anomaly and associated malignancies. Most existing literature consists of isolated case reports, which are subject to publication bias and do not allow for risk estimation or causal inference. Moreover, there are no specific guidelines for the management of CFRE, and surgical management is challenging due to complex vascular anatomy and fused parenchyma. In such patients, three-dimensional CT angiography helps delineate arterial supply and facilitates nephron-sparing surgery. Furthermore, initially, nephron-sparing surgery was evaluated, but it was not feasible due to tumor infiltration of more than 50% of both renal units and the complex, shared vascular anatomy, which increased the risk of incomplete resection and loss of residual renal function; thus, total nephrectomy was deemed oncologically and technically appropriate. Postoperatively, the patient was managed with hemodialysis and referred for renal transplantation, with follow-up focusing on metabolic stability and transplant eligibility.

## Conclusions

CFRE is a rare congenital anomaly that is often detected incidentally during investigation for other medical concerns that may rarely harbor renal malignancies. Although typically asymptomatic, its complex anatomical configuration poses unique diagnostic and therapeutic challenges, particularly when associated with RCC. Our case highlights the importance of maintaining a high index of suspicion and carefully evaluating atypical renal anatomy when interpreting imaging studies. Radiologists play a critical role in identifying this anomaly and accurately characterizing associated tumors. Detailed imaging evaluation of renal vasculature and tumor extent is essential for optimal surgical planning, particularly when nephron-sparing approaches are considered. Therefore, preoperative imaging provides a comprehensive roadmap to minimize intraoperative complications and ensure adequate oncological control. Overall, our report emphasizes the need for a multidisciplinary approach to optimize patient-associated clinical outcomes. However, our findings should be interpreted with caution due to the single-case nature of the study, even our patient did not report any adverse events, still lack of long term follow-up, and notably absence of detailed histopathological data.
